# Comparison of Effectiveness and Safety of Apixaban, Dabigatran, and Rivaroxaban in Patients With Valvular Atrial Fibrillation: A Network Meta-Analysis of Randomized-Control Trials and Observational Studies

**DOI:** 10.7759/cureus.57656

**Published:** 2024-04-05

**Authors:** Huria Huma, Anurag Rawat, Mandeep Kaur, Omkar Jha, Fahad Shaukat Gill, Merid Moqattash, Calvin R Wei, Danish Allahwala

**Affiliations:** 1 Cardiology, Glenifield General Hospital, Leicester, GBR; 2 Interventional Cardiology, Himalayan Institute of Medical Sciences, Dehradun, IND; 3 Internal Medicine, Hospital Corporation of America (HCA) Florida Capital Hospital, Tallahassee, USA; 4 Medicine, Health Foundation Nepal, Kathmandu, NPL; 5 Medicine, Shalamar Medical and Dental College, Lahore, PAK; 6 Medicine, Pecs Medical University, Pecs, HUN; 7 Research and Development, Shing Huei Group, Taipei, TWN; 8 Nephrology, Fatima Memorial Hospital, Karachi, PAK

**Keywords:** systematic review and meta-analysis, valvular atrial fibrillation, rivaroxaban, dabigartan, apixaban

## Abstract

The objective of this network meta-analysis was to assess the efficacy and safety of apixaban, dabigatran, rivaroxaban, and edoxaban in patients diagnosed with atrial fibrillation and valvular heart disease. A comprehensive search was conducted across various electronic databases, including PubMed, Embase, and Web of Science, from inception to February 15, 2024. The search strategy utilized a combination of medical subject headings (MeSH) terms and relevant keywords related to valvular heart disease, atrial fibrillation, anticoagulant therapy, and study design, such as randomized controlled trials and observational studies. The outcomes evaluated in this analysis comprised the incidence of stroke or systemic embolism (SE), as well as the occurrences of major bleeding events. A total of 10 studies were incorporated into this meta-analysis, encompassing 40,662 participants. Of these, 12,385 received apixaban, 2,829 received dabigatran, 13,662 received rivaroxaban, 2,582 received edoxaban, and 9,202 received warfarin. The duration of follow-up in the included studies ranged from 3 to 54 months. Among the four direct oral anticoagulants (DOACs) studied, apixaban demonstrated a significant reduction in the risk of stroke or SE when compared to other DOACs and warfarin, highlighting its efficacy in patients with atrial fibrillation and valvular heart disease. Additionally, apixaban exhibited a lower risk of major bleeding events, further emphasizing its favorable safety profile compared to the other agents assessed. In conclusion, our findings suggest that apixaban may be more effective and safer than other DOACs and warfarin in this patient population. However, additional studies are warranted to compare the various DOACs in this cohort to identify the optimal treatment strategy for preventing adverse outcomes.

## Introduction and background

Direct oral anticoagulants (DOACs) have been developed as a substitute for vitamin K antagonists (VKAs) and have emerged as the preferred treatment for atrial fibrillation (AF) in the general populace (Class I, Level of Evidence: B), as well as for the prophylaxis or treatment of deep vein thrombosis and pulmonary embolism [1]. Previous studies in non-valvular AF using DOACs have demonstrated their non-inferiority compared to warfarin use and, in some trials, superiority [2]. Patients with valvular heart disease constitute a distinct subset within the broader population of individuals with AF, encountering unique challenges and considerations in managing their condition. Valvular heart disease encompasses a spectrum of structural abnormalities affecting the heart valves, such as stenosis, regurgitation, or a combination thereof. The incidence of AF with valvular heart disease is on the rise, and the coexistence of both conditions is linked to an elevated risk of thromboembolism [3,4]. Despite the established effectiveness of anticoagulant therapy in reducing stroke risk in AF patients, uncertainty persists regarding the optimal anticoagulant choice for those with concurrent valvular disease.

The pivotal trials comparing DOACs to warfarin in patients with AF have enrolled only a limited number of individuals with valvular AF, with those who have undergone prior bioprosthetic valve replacement and/or repair being excluded from the primary analyses [5,6]. In patients with non-valvular AF, the non-inferiority of direct oral anticoagulants (DOACs) compared to warfarin has already been established [7,8]; hence, DOACs are widely adopted in routine clinical practice due to their avoidance of routine anticoagulation monitoring or dose adjustment [9]. DOACs have also proven effective as an alternative to warfarin in AF patients with valvular heart disease [10]. Current guidelines advocate for DOACs as the preferred initial therapy in AF patients with a bioprosthetic valve alongside warfarin [11].

Three DOACs have received approval, namely apixaban, dabigatran, and rivaroxaban. In the pivotal randomized controlled trials (RCTs) leading to their approval, each NOAC was assessed against warfarin. However, direct head-to-head RCTs comparing NOACs are currently unavailable, and indirect comparisons in network meta-analyses based on RCT data are constrained by variations in trial methodologies and participant demographics. Furthermore, there is a lack of network meta-analysis comparing apixaban, dabigatran, and rivaroxaban, specifically in patients with valvular heart disease. As a result, there is insufficient randomized evidence to aid clinicians in selecting an anticoagulant in clinical practice. Hence, we are conducting this network meta-analysis to evaluate the effectiveness and safety of apixaban, dabigatran, rivaroxaban, and edoxaban in patients with valvular heart disease.

## Review

Methodology

A comprehensive search was carried out across electronic databases, encompassing PubMed, Embase, and Web of Science, spanning from inception to February 15, 2024. The search strategy employed a blend of medical subject headings (MeSH) terms and pertinent keywords about valvular heart disease, atrial fibrillation, anticoagulant therapy, and study design (such as randomized controlled trials and observational studies). Supplementary studies were identified through manual perusal of reference lists from relevant articles and clinical trial registries. The search was restricted to articles published exclusively in the English language. Two authors independently searched, and any disparities in the search strategy process were resolved through discussion.

Study Inclusion Criteria

Studies were deemed eligible if they satisfied the following criteria: (1) inclusion of participants diagnosed with valvular heart disease, encompassing conditions such as mitral stenosis, mitral regurgitation, aortic stenosis, or aortic regurgitation, with or without coexisting atrial fibrillation; (2) comparison of DOACs either with VKA or with one another; (3) reporting of pertinent clinical outcomes, such as stroke or SE, major bleeding, myocardial infarction, or all-cause mortality; (4) classification as randomized controlled trials or cohort studies; (5) availability of adequate data for inclusion in meta-analysis. Studies in which specific DOACs were not analyzed were excluded. Additionally, reviews, editorials, animal studies, and those lacking a comparison group were also excluded.

All identified articles were imported into EndNote X9. Following the removal of duplication, two reviewers independently conducted initial screening based on titles and abstracts. The full text of all potentially eligible articles was obtained and evaluated against pre-defined inclusion and exclusion criteria. Any discrepancies in the study selection process were resolved through discussion.

Data Extraction and Quality Assessment

Two reviewers independently conducted data extraction using a standardized form created in Microsoft Excel. The extracted information encompassed study particulars (author, publication year, study design), participant demographics (age, gender), baseline comorbidities (hypertension, diabetes), details of intervention (anticoagulant type, dosage), and outcomes of interest (incidence of stroke or SE, and occurrences of major bleeding events). Any inconsistencies were resolved through mutual agreement or by seeking input from a third reviewer. The quality assessment of the included studies was performed using the Cochrane Risk of Bias Assessment.

Data Analysis

The data analysis strategy entailed conducting a network meta-analysis using RStudio software to assess the comparative effects of various interventions on the risk of stroke, SE, and major bleeding events. A random-effects model was employed to estimate point estimates along with 95% confidence intervals for the risk ratio (RR). Additionally, Surface Under the Cumulative Ranking (SUCRA) scores were computed to ascertain the likelihood of each treatment being the most effective. A comparison of direct comparison RRs was conducted against those derived from the network meta-analysis. Pairwise comparisons among the four direct oral anticoagulants (DOACs) concerning all-cause mortality and major bleeding events were presented alongside corresponding 95% confidence intervals. The analysis strategy aimed to offer comprehensive insights into treatment effects while addressing any potential limitations inherent in the data.

Results

**Figure 1:**
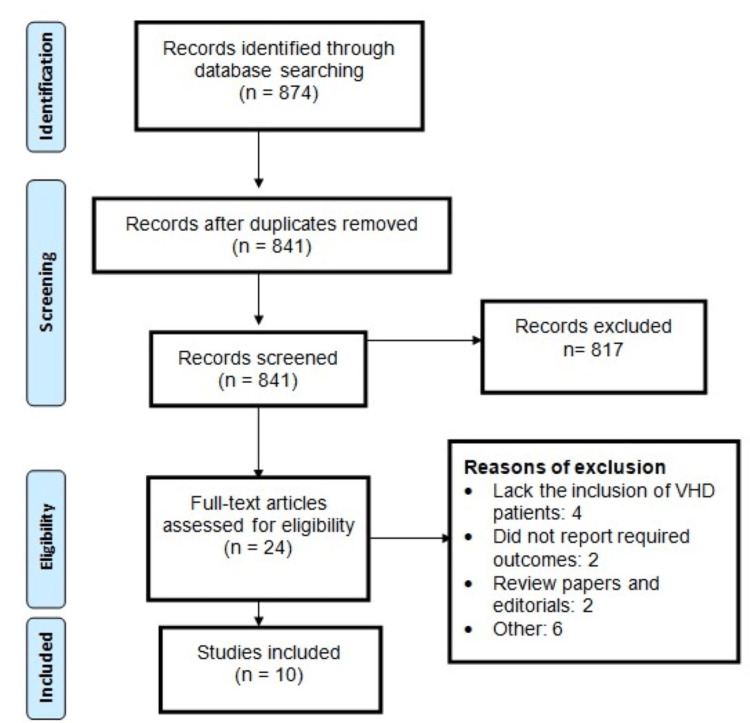
PRISMA flowchart of study selection

**Figure 2:**
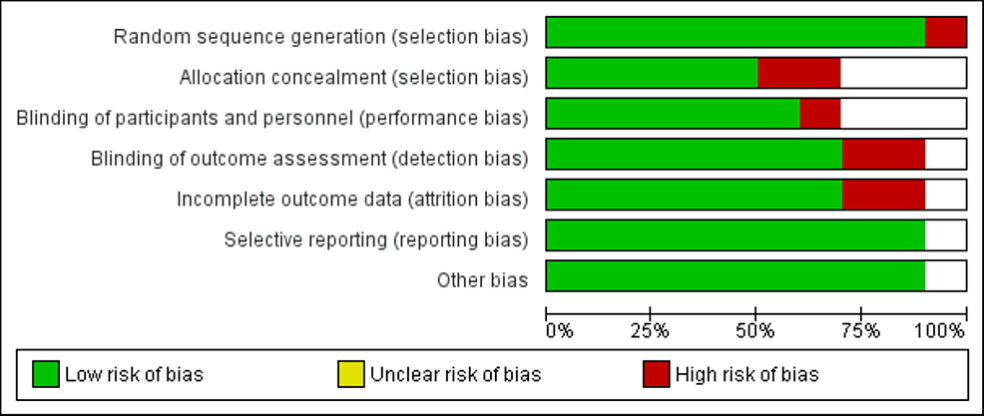
Risk of bias assessment of included studies

The literature selection process is outlined in Figure 1. In total, 874 studies were identified through comprehensive online database searches. Initial screening involved assessing 841 records, followed by a full-text evaluation of 24 studies. Subsequently, 10 studies were deemed suitable for inclusion in this network meta-analysis. Table 1 provides a summary of the characteristics of these selected studies. The majority of them were randomized controlled trials (RCTs). The analysis encompassed a total of 40,662 participants, with 12,385 receiving apixaban, 2,829 receiving dabigatran, 13,662 receiving rivaroxaban, 2,582 involved in edoxaban, and 9,202 receiving warfarin. The follow-up durations of the included studies ranged from 3 to 54 months. The primary efficacy outcomes assessed in most of the studies were stroke and SE while bleeding events were frequently evaluated as safety outcomes. All the included studies reported results spanning from 2013 to 2022. Figure 2 presents the risk of bias assessment in the included studies. 

**Table 1:** Characteristics of the included studies RCT: Randomized control trial; UK: Unknown

First Author	Year	Setting	Study Design	Groups	Sample size	Follow-up
Avezum et al. [12]	2014	Multicenter	RCT	Apixaban	2438	21.8 Months
				Warfarin	2370	
Breithardt [13]	2014	Multicenter	RCT	Rivaroxaban	940	36 Months
				Warfarin	1005	
Caterina et al. [14]	2017	Multicenter	RCT	Edoxaban	1869	33.6 Months
				Warfarin	955	
Connolly et al. [15]	2022	Multicenter	RCT	Rivaroxaban	2275	54 Months
				Warfarin	2256	
Dawwas et al. [16]	2022	Single Center	Observational	Apixaban	9947	12 Months
				Rivaroxaban	9947	
Duraes et al. [17]	2016	Single Center	RCT	Dabigartan	15	3 Months
				Warfarin	12	
Eikelboom et al. [18]	2013	Multicenter	RCT	Dabigartan	168	3 Months
				Warfarin	84	
Ezekowitz et al. [19]	2016	Multicenter	RCT	Dabigartan	2646	UK
				Warfarin	1304	
Guimaraes et al. [20]	2020	Multicenter	RCT	Rivaroxaban	500	12 Months
				Warfarin	505	
Mieghem et al. [21]	2021	Multicenter	RCT	Edoxaban	713	36 Months
				Warfarin	713	

Stroke or Systemic Embolism (SE)

**Figure 3:**
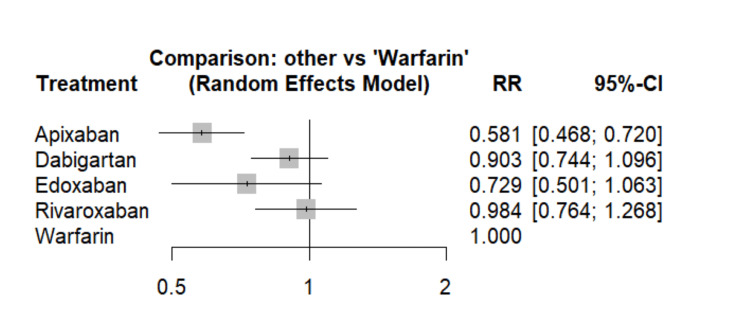
Comparison of DOACs versus Warfarain in the prevention of stroke and SE RR: Risk ratio; CI: Confidence interval Sources: References [12-21]

This network meta-analysis incorporated a total of 10 studies to assess the risk of stroke or SE across various anticoagulant treatments. Apixaban demonstrated a significant association with a reduced risk of stroke, or SE when compared to vitamin K antagonists (VKA) in patients with valvular heart disease. Specifically, the use of apixaban was linked to a 48% reduction in stroke or SE events, as depicted in Figure 3. Conversely, while the risk of stroke or SE events appeared lower in the dabigatran, edoxaban, and rivaroxaban groups compared to warfarin, these differences did not attain statistical significance. The effect estimates obtained from direct pairwise comparisons closely mirrored those derived from the network meta-analysis, both in terms of direction and magnitude, as elucidated in Table 2. Apixaban emerged with the highest SUCRA score, signifying a substantial likelihood of greater effectiveness compared to warfarin in preventing stroke, or SE. Following apixaban, edoxaban exhibited the next highest SUCRA score, indicating a notable probability of superiority over warfarin, followed by dabigatran and rivaroxaban, respectively.﻿

**Table 2:** Comparison of effect estimates from direct pair-wise comparisons and network meta-analysis for stroke or SE NMA: Network meta-analysis; RR: Risk ratio; CI: Confidence interval; SUCRA: Surface Under the Cumulative Ranking curve

Treatment	Pair-wise Meta-Analysis, RR (95% CI) versus Warfarin	NMA, RR (95% CI) versus Warfarin	SUCRA
Apixaban	0.59 (0.46 to 0.74)	0.58 (0.47 to 0.72)	95.75%
Dabigartan	0.90 (0.74 to 1.10)	0.90 (0.74 to 1.10)	41%
Rivaroxaban	0.97 (0.72 to 1.30)	0.98 (0.76 to 1.27)	23.25%
Edoxaban	0.73 (0.50 to 1.06)	0.73 (0.50 to 1.06)	72.25%

Major Bleeding Events

**Figure 4:**
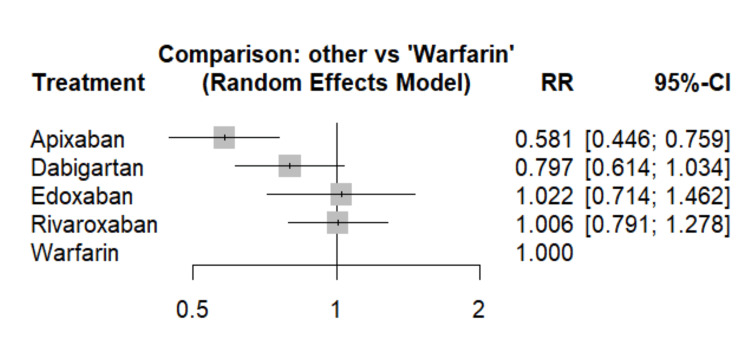
Comparison of major bleeding events between different DOACs and warfarin RR: Risk ratio; CI: Confidence interval Sources: References [12-21]

The primary analysis of major bleeding was based on an evidence network comprising 10 studies, encompassing four treatments alongside warfarin. As illustrated in Figure 4, apixaban demonstrated a statistically significant decrease in the risk of major bleeding when compared to warfarin. Dabigatran also showed a reduction in major bleeding events compared to warfarin, albeit not reaching statistical significance. Conversely, no statistically significant disparities in major bleeding risk were observed between warfarin and either edoxaban or rivaroxaban. The effect estimates from direct pairwise comparisons closely mirrored those obtained from the network meta-analysis in terms of both direction and magnitude, as presented in Table 3. Apixaban exhibited the highest SUCRA score (97.50%), indicating a strong likelihood of superior effectiveness, followed by dabigatran and rivaroxaban.

**Table 3:** Comparison of effect estimates from direct pair-wise comparisons and network meta-analysis for major bleeding events NMA: Network meta-analysis; RR: Risk ratio; CI: Confidence interval; SUCRA: Surface Under the Cumulative Ranking curve

Treatment	Pair-wise Meta Analysis, RR (95% CI) versus Warfarin	NMA, RR (95% CI) versus Warfarin	SUCRA
Apixaban	0.62 (0.45 to 0.84)	0.58 (0.45 to 0.76)	97.50%
Dabigartan	0.80 (0.61 to 1.03)	0.80 (0.61 to 1.03)	69.25%
Rivaroxaban	0.96 (0.74 to 1.26)	1.01 (0.79 to 1.28)	29.25%
Edoxaban	1.02 (0.71 to 1.46)	1.02 (0.71 to 1.46)	25.50%

Pairwise Comparison Among Different DOACs 

**Figure 5:**
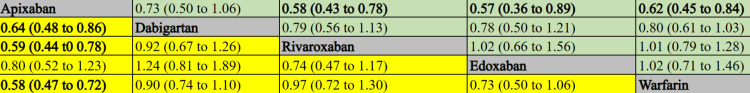
Below the diagonal, in yellow, are the relative risks (RRs) from network meta-analyses concerning stroke or systemic embolism, while above the diagonal, in green, are the RRs for major bleeding for all pairwise comparisons. Significant findings are highlighted in bold. To derive RRs for comparisons in the opposite direction, reciprocals must be calculated. All values are presented as relative risk (95% confidence interval)

Figure 5 presents a summary of all pairwise comparisons derived from the Bayesian random-effects network meta-analysis. Regarding pairwise comparisons among four different direct oral anticoagulants (DOACs) for stroke or SE, apixaban demonstrated a significant association with reduced risk compared to dabigatran and rivaroxaban. However, no significant difference was observed between apixaban and edoxaban. Conversely, no significant difference was found between dabigatran, rivaroxaban, and edoxaban in terms of the risk of stroke or SE. In terms of major bleeding events, apixaban exhibited a significant association with a reduced risk of bleeding events compared to edoxaban and rivaroxaban. However, the difference was not significant in the comparison between apixaban and dabigatran. Additionally, dabigatran showed a significantly reduced risk of bleeding events compared to edoxaban and rivaroxaban; however, the difference was not statistically significant.

Discussion

We have identified 10 studies that compare four distinct DOACs alongside warfarin concerning the prevention of all-cause stroke, or SE, and major bleeding among patients with valvular heart disease. To our knowledge, this marks the inaugural systematic review and network meta-analysis endeavoring to amalgamate the available advantages and drawbacks of newer oral anticoagulants for averting stroke and major bleeding occurrences in patients grappling with valvular heart disease. A recent meta-analysis by Batool et al. [10], encompassing five randomized controlled trials (RCTs), concluded that DOACs exhibit greater efficacy than warfarin in preventing stroke and major bleeding events. Nonetheless, our current meta-analysis encompasses recently published RCTs as well as observational studies. Moreover, we conducted pairwise comparisons among DOACs to gauge the comparative effectiveness of the four DOACs in preventing stroke, SE, and major bleeding events. Valvular heart disease represents a prevalent condition in both developed and developing nations, imposing a substantial burden on constrained healthcare resources. Unlike other cardiovascular ailments, there is a paucity of randomized clinical trials scrutinizing the impacts of medical interventions in patients with valvular heart disease [11]. Additionally, there is a dearth of studies directly comparing DOACs in the context of valvular heart disease.

For patients at risk of stroke and SE, the utilization of apixaban has demonstrated significant advantages, as it has been associated with notable reductions in the incidence of stroke or SE when compared to conventional therapy involving warfarin. Conversely, patients treated with dabigatran, rivaroxaban, and edoxaban have also exhibited a decreased risk of stroke or SE compared to those receiving warfarin. However, in these instances, no statistically significant disparity was observed between the efficacy of the alternative treatments and that of warfarin therapy. Although all these anticoagulant medications have shown promising outcomes in diminishing the risk of stroke or SE, apixaban has emerged as particularly effective in achieving significant risk reduction when compared to warfarin. Apixaban boasts a predictable pharmacokinetic profile, obviating the need for routine monitoring or dose adjustments based on international normalized ratio (INR) levels, unlike warfarin. This attribute simplifies its administration and may contribute to more consistent anticoagulation levels [22]. The ARISTOTLE trial [12] provided scant evidence of a differential effect of apixaban versus warfarin in reducing stroke and SE, major bleeding, and mortality rates in patients with and without VHD. Similarly, a post hoc analysis of the ROCKET AF trial [13] yielded little evidence of a differential effect of rivaroxaban versus warfarin in reducing stroke and SE in patients with and without VHD.

Furthermore, it was determined that apixaban presented a diminished risk of major bleeding events compared to warfarin, emphasizing its favorable safety profile. Similarly, patients receiving dabigatran, rivaroxaban, and edoxaban also exhibited a decreased risk of major bleeding events in comparison to those undergoing warfarin treatment. However, the distinction in efficacy between these alternative treatments and warfarin did not achieve statistical significance in these scenarios. Remarkably, in terms of preventing major bleeding events, apixaban emerged as the most effective among all other DOACs. These observations underscore the significance of considering both efficacy and safety profiles when determining anticoagulant therapies for patients with atrial fibrillation.

As of present, warfarin remains the primary anticoagulant utilized for valve surgery and VHD [3]. Traditionally, mechanical valves have necessitated warfarin as the exclusive treatment option, while bioprosthetic valves typically require adjunctive antiplatelet therapy [23]. Numerous ongoing studies are evaluating the efficacy of novel oral anticoagulants (NOACs) in managing VHD. Despite the limited available evidence, NOACs may be considered for select patients with VHD, provided they are not contraindicated. Ultimately, the decision to prescribe NOACs to patients rests with physicians, who must carefully weigh the risks and benefits. Physicians should tailor their choice of medication based on a personalized assessment of the valvular pathology and the patient's functional status, devising a management plan that best suits the individual's needs.

It is worth noting that patients with VHD have frequently been underrepresented or excluded from randomized controlled trials assessing the efficacy or safety of oral anticoagulants. This exclusion has been partly influenced by uncertainties regarding whether the pathogenesis of thromboembolism differs between patients with atrial fibrillation (AF) with and without VHD [24]. Moreover, randomized controlled trials and clinical practice guidelines have adopted varying definitions of valvular AF, resulting in differences in inclusion and exclusion criteria across trials [16]. These discrepancies in defining valvular AF also impact clinical practice. In the future, there is a necessity for more studies comparing different DOACs to determine the optimal treatment for patients with VHD.

Study Limitations

The present meta-analysis possesses several limitations. First, the majority of our findings stem from data derived from post-hoc analyses of large randomized controlled trials (RCTs). The populations encompassed within the studies included in our analysis exhibit a degree of heterogeneity involving the analysis of various medications. Consequently, combined outcome analyses may either overestimate or underestimate the observed benefits. Second, we were unable to conduct subgroup analyses based on the specific types of VHD. Therefore, there is a pressing need for additional studies that compare DOAC among themselves and against warfarin to ascertain the optimal treatment regimen for patients with VHD.

## Conclusions

In conclusion, our meta-analysis synthesized data from 10 studies, delineating the efficacy and safety profiles of direct oral anticoagulants (DOACs) and warfarin in valvular heart disease (VHD) management. Apixaban demonstrated a significant reduction in stroke or systemic embolism (SE) risk compared to other DOACs and warfarin, underscoring its efficacy. Furthermore, apixaban exhibited a lower risk of major bleeding events, emphasizing its favorable safety profile. However, limitations include heterogeneous study populations and the absence of subgroup analyses based on VHD type. Future research should focus on comparative studies to optimize treatment selection for VHD patients, considering both efficacy and safety.
